# Association between COX-2 rs 6681231 Genotype and Interleukin-6 in Periodontal Connective Tissue. A Pilot Study

**DOI:** 10.1371/journal.pone.0087023

**Published:** 2014-02-13

**Authors:** Francisco Mesa, Francisco O’Valle, Manfredi Rizzo, Francesco Cappello, Nikos Donos, Mohamed Parkar, Navidah Chaudhary, Francesco Carini, Ricardo Muñoz, Luigi Nibali

**Affiliations:** 1 Department of Periodontics, School of Dentistry, University of Granada, Granada, Spain; 2 Departments of Pathology, School of Medicine and Biopathology and Medicine Regenerative Institute (IBIMER), University of Granada, Granada, Spain; 3 Department of Internal Medicine, University of Palermo, Palermo, Italy; 4 Department of Experimental Medicine and Clinical Neuroscience, Section of Human Anatomy, University of Palermo, Palermo, Italy; 5 Periodontology Unit and Department of Clinical Research, University College London (UCL) Eastman Dental Institute and Hospital, London, United Kingdom; 6 Euro-Mediterranean Institute of Science and Technology, Palermo, Italy; 7 Andalusian Service of Public Health, Granada, Spain; Boston University, United States of America

## Abstract

**Objectives:**

The aim of this pilot study was to investigate associations between IL-6 and COX-2 expression in gingival biopsies and both clinical diagnosis and genotypes in the IL-6 and COX-2 genes.

**Design:**

A case-control study included 41 gingival biopsies obtained from Caucasian patients grouped according to clinical diagnosis of gingival health (n = 10), gingivitis (n = 15) or chronic periodontitis (n = 16). Immunohistochemistry analyses were performed to determine COX-2 expression in lamina propria, IL-6 expression in lamina propria and gingival epithelium and level of inflammatory cell infiltrate. Individual DNA was extracted and genotyped by real-time PCR for *IL6* SNPs rs 2069827 and rs 2069825 and for *COX-2* rs 6681231.

**Results:**

The percentage of cellular COX-2 expression was associated with the extent of periodontal disease (Arbes index p = 0.026) and inflammatory infiltrate (p<0.0001). No association was observed between *IL6* haplotypes and cells positive to IL-6 or COX-2 in gingival tissues. The *COX-2* rs 6681231 was associated with cells positive to IL-6 in the connective tissue (p = 0.032).

**Conclusions:**

COX-2 expression in gingival tissues may be a marker of periodontal disease severity. *COX-2* rs 6681231 may be associated with IL-6 local production in gingival tissues.

## Introduction

The systemic immune response, genetic factors and environmental factors affect the risk of developing periodontitis [1], [2]. In recent years, studies have demonstrated that elevated levels of a variety of inflammatory biomarkers [3], [4] and genetic variants of some cytokines confer susceptibility to periodontitis [5]–[7].

Interleukin-6 (IL-6) is a cytokine produced by lymphocytes, monocytes, fibroblasts and endothelial cells, with functions in the systemic inflammatory response and on regulation of the acute phase response [8]. In particular, IL-6 is able to stimulate the synthesis of all the acute phase proteins involved in the inflammatory response C-reactive protein, serum amyloid A, fibrinogen, α1-chymotrypsin and haptoglobin [9]. Cyclooxygenase-2 (COX-2) is an enzyme involved in the conversion of arachidonic acid to prostaglandins, with a consequent important role in inflammatory responses, also in the periodontium [10], [11]. COX-2 expression is induced by cytokines [12] and, on the other hand, prostaglandins are important regulators of IL-6 production by gingival fibroblasts [13]. Therefore, within the multitude of actions on a wide range of cells and matrix structures in complex organ systems, IL-6 and COX-2 have the potential to influence one another.

A large number of scientific papers investigated the role of gene variants (polymorphisms) in host responses in periodontitis, and in the progression of the disease [14]. *IL6* polymorphisms and haplotypes have been shown to affect IL-6 transcription and therefore circulating interleukin-6 levels [15]–[17]. A recent meta-analysis of the *IL6* −174 polymorphisms did not show any association for this polymorphism with chronic periodontitis [14]. However, an association was found between IL-6 −174 and aggressive periodontitis in a separate meta-analysis [18]. Similarly, genetic regulation of COX-2 production has been associated with periodontitis [19]. Recently, a large study in Caucasians showed an association between *COX-2* rs 6681231 genetic polymorphism and presence of aggressive periodontitis [20].

The objective of this pilot study was to investigate the association between IL-6 and COX-2 expression in gingival biopsies and both clinical presentation (disease severity) and genetic polymorphisms in the *IL6* and *COX-2* genes.

## Materials and Methods

### Ethical considerations

All participants signed informed consent, and the study was conducted in accordance with the Helsinki declaration (version 2002) and approved by the ethics/research committee of the University of Granada (ref 04/072011).

### Clinical examination and subject selection

A case-control and analytic study was conducted in 41 adults requiring tooth extraction for caries, tooth fracture, endodontic failure or tooth mobility (>1 mm in buccal-lingual direction and cause of discomfort). Inclusion criteria were the same for all three groups: Caucasian ethnicity, age >18 years and the presence of at least 6 teeth. Exclusion criteria were periodontal treatment in the previous year, antibiotics or anti-inflammatory treatment in the previous 2 months and self-reported diagnosis of diabetes. Demographic (age, gender) and clinical data were collected by a calibrated examiner (R.M.). Gingival inflammation was assessed using the gingival bleeding index [21] and probing depth and loss of clinical attachment were determined using a PCPUNC15 periodontal probe (1 mm increments) (University of North Carolina, Hu-Friedy, Chicago, IL), at six sites per tooth (mesiovestibular, vestibular, distovestibular, mesiolingual, lingual, and distolingual). CP extent was classified by the percentage of sites with a loss of attachment ≥3 mm [22]. Following periodontal examination, participants were grouped according to clinical diagnosis of chronic periodontitis (CP), gingivitis (G), or gingival health (control = C). For inclusion to this study:

- Chronic Periodontitis (CP) was classified by the presence of at least 1 site with clinical attachment level (CAL) ≥3 mm and at least 1 site with probing pocket depth (PPD) of ≥6 mm
- Gingivitis (G) was defined by any gingival bleeding on probing (>0%) [21] and absence of PPD ≥3 mm
- Gingival health (C) was defined as no gingival bleeding on probing [21] and absence of PPD ≥3 mm.

Each patient contributed with one site to the study sampling procedures. All patients were treated with tooth extraction (and periodontal treatment if appropriate) at the School of Dentistry, University of Granada (Spain) from September 2010 to June 2011. Immediately before the tooth extraction, the same clinician (F.M.) used a scalpel to take a single biopsy from the mesial or distal gingival papilla of each patient after anesthetization with 2% mepivacaine. In CP patients, biopsies were obtained before tooth extraction at sites with probing depth of ≥6 mm with bleeding on probing and evidence of bone loss on periapical X-ray. In gingivitis patients, biopsies were obtained before tooth extraction at sites with bleeding on probing and PPD <3 mm.

### Examiner’s reproducibility

The examiner (author R.M.) was trained and calibrated on 10 patients probed twice before conducting periodontal examinations. Intra-examiner reliability was assessed by using the kappa statistic, which was equal to 0.78, evidencing a high degree of consistency in the observations. The examiner was also calibrated against a ‘gold-standard examiner’ (author F.M.) showing good concordance, with a kappa statistics of 0.82.

### Immunohistochemistry analyses

- COX-2 expression: Paraffin-embedded sections were dewaxed, hydrated, and heat-treated in 1 mM EDTA buffer pH 8 for antigenic unmasking in a PT module (Thermo Fisher Scientific Inc., Waltham, MA) at 95°C during 20 min. Sections were incubated for 30 min at room temperature with anti-COX-2 (clone SP21) rabbit monoclonal antibody diluted 1∶50 (Master Diagnóstica, Granada, Spain) in order to identify cell inflammatory expression. The immunohistochemistry study was done on an automatic immunostainer (Autostainer480, Thermo Fisher Scientific Inc) using the polymer-peroxidase-based method, followed by development with diaminobenzidine (Master Diagnóstica). Appropriate positive controls, as well as non-immune serum for negative controls, were run concurrently. Nuclear counterstaining was done using hematoxylin (Master Diagnóstica). The immunohistochemistry results were calculated as percentage of cells positive to COX-2 and semi-quantitatively using a 0 to 3 scale (0, absence; 1, mild [<10% of cells positives]; 2, moderate [10 to 25%]; 3, severe [>25%]).
- IL-6 expression: Five-micrometre thick sections were exposed to immunohistochemistry staining using the polyclonal antibody against human IL-6 (sc-130326), Santa Cruz Biotechnology Inc., Germany) as described before [23]. The histological measurements were performed using a microscope (Olympus BX50, Best Scientific LTD, Wroughton, UK) equipped with an imaging system (Image Pro-PLUS 4.5, Media Cybernetics, Bethesda, USA). Each slide was categorised into two zones: infiltrated connective tissue (ICT) and periphery of the ICT (non-ICT). A 400-point lattice was superimposed over the tissue area at a magnification of ×400, and the number of cross-points on positive cells was counted in duplicate and expressed as a percentage of the tissue area related to the total number of points [23].

### Histological evaluation of the inflammatory infiltrate

The presence of inflammatory cells (namely lymphocytes, granulocytes, monocytes/macrophages and plasma cells) in the specimens was detected microscopically and recorded. The inflammatory infiltrate level was calculated microscopically in a semi-quantitatively manner on a 4-point scale: 0, absence of any such inflammatory cells: 1, mild [<10% of lamina propria involved]; 2, moderate [10 to 25%]; 3, severe [>25%]).

### Genetic analyses

DNA was extracted from histological sections with a commercially-available kit (‘Recover All Total Nucleic Acid Isolation’, Life Technologies, Paisley, UK) and genotyped for two polymorphisms in the *IL6* gene promoter region using the Applied Biosystems 7300 Real Time PCR System as previously described [24]. The studied polymorphisms were rs 2069827 and rs 2069825 in the *IL6* gene and rs 6681231 in the *COX-2* gene. Homozygous subjects for rs 2069827 G allele and rs 2069825 C allele (no deletion) were defined as H+, while all other subjects were H- [25]. Duplicates were added to each plate to test for error rates. Each clinical sample was anonymously coded, so the immunohistochemistry and genetic analyses were performed independently and blind to clinical diagnosis.

### Statistical analysis

A specific computer program (SPSS-Windows 20.0 program SPSS IBM Inc, Chicago, IL) was used for the statistical analyses. Data on IL-6 and COX-2 expression in gingival tissues were plotted and their normal distribution was confirmed. After descriptive analysis, non-parametric tests (Mann-Whitney U-test, Kruskal Wallis) and Spearman’s Rho coefficient were used to evaluate the associations between IL-6 and COX-2 expression in gingival tissues (primary outcomes) and clinical diagnosis and IL-6 and COX-2 SNPs (explanatory variables). No formal sample size calculation was performed due to the pilot nature of the study. The potential effect of confounders such as gender and age was explored. The α value was set at 0.05. The tests used are reported in table footnotes.

## Results

The study included 41 gingival biopsies obtained from 16 Caucasian patients with CP, 15 with G and 10 with gingival health. Table 1 shows inter-group comparisons of demographic and clinical variables (bleeding on probing) and genetic variables (*IL6* and *COX-2*). Table 2 shows histological results in patients divided by clinical diagnosis: COX-2 immunohistochemistry expression in lamina propria (positive plasma cells and monocytes) and IL-6 expression in lamina propria and gingival epithelium. The mean ± S.D. of percentage of sites with a loss of attachment ≥3 mm (Arbes index) in the CP group was 24.8±18.0. The mean probing pocket depth at the sites where the biopsy specimens were taken in CP patients was 7.12±2.65 mm.

**Table 1:** Comparison between groups of studied periodontal, immunohistochemistry and genetic variables (n = 41).

Variables	Control (n = 10)	Gingivitis (n = 15)	Periodontitis (n = 16)	p-value
Age (years)	35.2±14.7	46.6±17.9	44.9±9.4	0.112^b^
Gender (male)	7 (77.8%)	6 (50.0%)	11 (68.8%)	0.469^a^
Bleeding on probing %*	0	10.2±6.9	60.6±25.2	<0.001^c^
IL-6 H+	5 (55.6%)	5 (45.5%)	4 (40.0%)	0.790^a^
IL-6 H−	4 (44.4%)	6 (54.5%)	6 (60.0%)	
COX-2 genotype GC	1 (12.5%)	5 (38.5%)	6 (46.2%)	0.280^a^
COX-2 genotype CC	7 (87.8%)	8 (61.5%)	7 (53.8%)	

Continuous values are expressed as mean ± standard deviation;

a: Chi square test;

b: Kruskal-Wallis test;

c: Mann-Whitney U-test. Please note: Genotype results are available in 30/41 subjects for IL6 genotypes and in 34/41 subjects for COX-2 genotype.

**Table 2:** Medians and inter-quartile range of clinical (Arbes index) and histological results for subjects divided by clinical diagnosis.

Variable	Control (n = 10)	Gingivitis (n = 15)	Periodontitis (n = 16)	Comparisons between groups
Arbes index	−	−	18 (12–46)	−
Inflammatory cells detected	1 (10%)	15 (100%)	16 (100%)	<0.001^a^
Cox2 Plasma cells	0 (0–1)	12.5 (2.5–62.5)	40 (15–100)	0.009^a^
Cox2 Monocytes	1 (0–2)	10 (5–62.5)	40 (15–100)	0.009^a^
IL-6 connective tissue	12.25 (11–15)	10.75 (9–12.5)	13 (10–15)	0.346^b^
IL-6 epithelium	12.25 (7–18)	15.25 (14–20.5)	15 (9–20.5)	0.582^b^

a: Chi square test;

b: Kruskal-Wallis test.

### Immunohistochemistry outcomes

**Figure 1:**
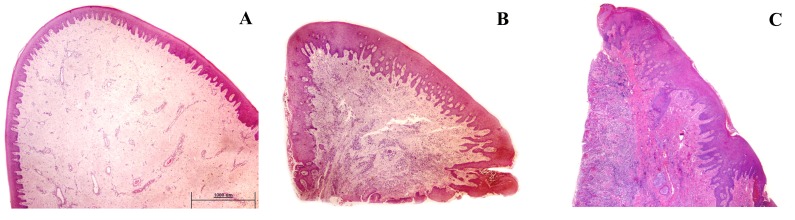
Panoramic microphotograph of gingival biopsies. A) Gingival health group; B) Gingivitis; C) Chronic periodontitis. (Hematoxylin-eosin, original magnification ×2). (Bar 1000 µm).

**Figure 2:**
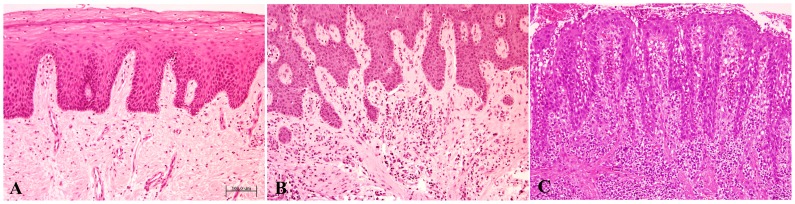
Gingival epithelium and lamina propria with variable chronic inflammatory infiltrate. A) Mild infiltrate in gingival health group; B) mild/moderate infiltrate in gingivitis; and C) severe infiltrate in sulcular epithelium in chronic periodontitis (Hematoxylin-eosin, original magnification ×10). (Bar 100 µm).

**Figure 3:**
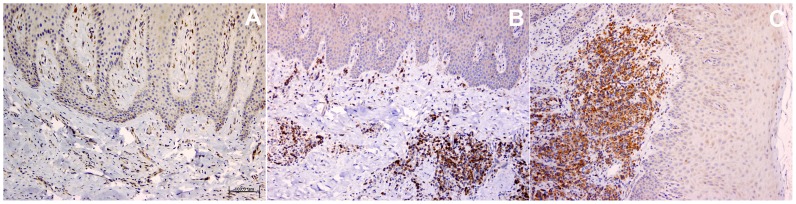
Representative microphotograph of COX-2 immunohistochemical expression (brown-stained) in gingival biopsies. A) Gingival health group; B) Gingivitis; C) Chronic periodontitis (Polymer-peroxidase-based method, original magnification ×10).

**Figure 4:**
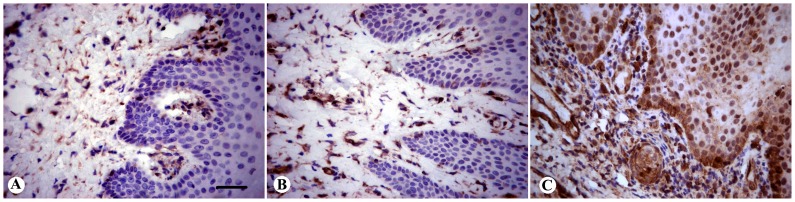
Representative microphotograph of IL-6 immunohistochemical expression (brown-stained) in gingival biopsies. A) Gingival health group; B) Gingivitis; C) Chronic periodontitis (original magnification ×40) (Bar 100 µm).

The clinical diagnosis was confirmed histopathologically, since inflammatory cells in the specimens were detected in only 1 out of 10 healthy, 15/15 G and 16/16 CP cases (see figures 1 and 2). The mean percentage of inflammatory cells in CP cases was lymphocytes 85% (50% of which were mature plasma cells), granulocytes 9%, and monocytes/macrophages 6% (data not presented). Figure 3 and 4 show immunohistochemistry expression of COX-2 and IL-6 respectively in the different study groups. The number of IL-6 positive cells by immunohistochemistry was similar across clinical diagnoses (CP, G, C). On the other hand, COX-2 expression in plasma cells and monocytes was associated with clinical diagnosis (both p = 0.009), with a gradient increase from C to G and to CP (Table 2). The percentage of cellular COX-2 expression was associated with disease severity measured as Arbes index (p = 0.026) and inflammatory infiltrate level (rho coefficient = 0.674, p<0.0001). A positive association was also detected between inflammatory infiltrate severity and immunohistochemistry expression of COX-2 in plasma cells and monocytes of gingival lamina propria (Rho = 0.569, p<0.0001; Rho = 0.565, p<0.0001, respectively). No associations were detected between immunohistochemistry outcomes and demographic factors (age and gender).

### Genetic outcomes

No error rates were detected in the genetic analyses. However, some of samples yielded a small concentration of DNA, which was not suitable for genetic analysis and was not scored (15/82 IL-6 SNPs and 7/41 COX-2 SNP, overall 17.9%). The Hardy-Weinberg equilibrium was satisfied for all three studied SNPs.

The *COX-2* rs 6681231 did not show any correlation with COX-2 expression at immunohistochemistry (p = 0.884) but was associated with cells positive to IL-6 in the connective tissue (Rho = 0.449, p = 0.032) (Table 3).

**Table 3:** COX-2 gingival expression (median and inter-quartile range) and IL-6 cells positive in the periodontal connective tissue (CT) and outside CT in subjects divided by COX-2 rs 6681231 genotypes.

		Immunohistochemistry results		
		COX %	IL-6 in CT	IL-6 outside CT
COX-2 rs 6681231 genotypes	CC (n = 22)	10 (5–50)	13.5 (11–16.5)*	13 (6.5–20.5)
	GC (n = 12)	10 (1–50)	10 (6.5–13)*	16.7 (13–25)

*P = 0.032 Mann-Whitney U test.

The *IL6* haplotype was not associated with the number of IL-6 positive cells at immunohistochemistry (lamina propria p = 0.224, epithelium p = 0.580, Mann-Whitney U-test) or plasma cells and monocytes COX-2 expression (p = 0.307, p = 0.134, p = 0.191, respectively). IL-6 connective and epithelium immunohistochemistry expression did not correlate with COX-2 expression total percentage (Rho = 0.01, p = 0.944; Rho = 0.01, p = 0.946), plasma cells (Rho = −0.06, p = 0.743; Rho = −0.01, p = 0.940) and monocytes (Rho = 0.009, p = 0.962; Rho = −0.02, p = 0.904).

## Discussion

This study showed a relationship between immunohistochemistry expression of COX-2 and periodontal pathology (gingivitis and chronic periodontitis). Furthermore, it produced initial evidence for a possible association between *COX-2* genotype rs 6681231 and local IL-6 expression (measured as number of periodontal connective tissue cells positive to IL-6 at immunohistochemistry).

There is strong evidence that genetic, as well as environmental factors, affect the onset and severity of periodontitis [1]. Despite the publication of several genetic association studies in the field of periodontitis, including recent GWAS (genome-wide association studies), there is considerable uncertainty as to which genetic variants predispose to periodontitis. *IL*6 gene promoter and *COX-2* polymorphisms are some of a few promising genetic variants studied in association with periodontitis in different populations [20], [26], [27], [28]. However, studies for independent validation of these findings and functional studies showing the effect of these genetic variants in inducing periodontal pathology are still lacking.

To the best of our knowledge, this is the first study conducted in a Caucasian population investigating the possible effect of *COX-2* and *IL6* haplotypes on local expression of COX-2 and IL-6 in the gingiva. Interestingly, all three studied patient groups (gingival health, gingivitis and chronic periodontitis) exhibited a similar average of IL-6 positive cells in the gingival biopsies, while COX-2 expression was significantly associated with periodontal disease severity. The association between COX-2 periodontal expression and periodontal pathology confirms a previous report from our group [29] and others [30], highlighting the role of this enzyme in periodontal destruction. However, it is not clear whether increased production of COX-2 may predispose to periodontitis or if, on the other hand, COX-2 increased expression is just a result of chronic exposure to periodontopathogenic bacteria.

In the present study, the rare allele frequency of *COX-2* polymorphism rs 6681231 (17.6%) was almost identical to the frequency reported in the HapMap CEPH Utah reference population of European ancestry (17.8%) (www.hapmap.org). The rare G allele, which was enriched in chronic periodontitis patients in the present study, was found to be associated with aggressive periodontitis in a large North-European Caucasian population [20]. This SNP was not associated with COX-2 expression in our sample, but showed an association with IL-6 cellular infiltrate in the connective tissue. Studies in Taiwanese and Chinese populations [19], [31] reported that the −765 C polymorphism of the COX-2 gene is associated with a decreased risk for periodontitis in these populations, especially in aggressive periodontitis. In contrast with this, functional analyses on COX-2 −765 polymorphism have reported that the C allele may reduce the COX-2 gene expression and consequently inhibit inflammatory responses [32], [33]. A possible reason for the discrepancy is that the clinical role of COX-2 in inflammation may be dual by initiating the process of inflammation and then later aiding in its resolution. The effects of COX-2 genotypes in the inflammatory response through variations in COX-production have been shown with relation to C-reactive protein [32] and IL-6 [34]. In particular, PEG2 is important in regulating IL-6 production and COX-2 inhibitors have been shown to be useful in controlling fibroblast IL-6 production. The effect of COX-2 is particularly important in gingival fibroblast IL-6 production [13], which would explain the association observed in our study with IL-6 positive cells in the connective tissue but not in the epithelial infiltrate. The lack of association between *COX-2* genetic polymorphisms and COX-2 gingival expression may be due to the pilot nature of this study, which might help in a sample size calculation for a future study investigating this outcome.

In conclusion, this study confirms a possible role for COX-2 in periodontal pathology and it is the first study to provide initial evidence that the COX-2 rs 6681231 genotype may affect IL-6 local production in gingival tissues. Limitations of this study are inherent to its pilot nature and include the small sample size, with the risk of type II error and the residual potential confounding effect of smoking. Therefore, the present findings need to be confirmed by larger investigations. If these results were confirmed, IL-6 may be considered an important mediator in the role of COX-2 polymorphisms in periodontal pathology.

## References

[1] Van Dyke TE, Sheilesh D (2005) Risk factors for periodontitis. J Int Acad Periodontol 7: 3–7. 15736889 PMC1351013

[2] Agrawal AA, Kapley A, Yeltiwar RK, Purohit HJ (2006) Assessment of single nucleotide polymorphism at IL-1A+4845 and IL-1B+3954 as genetic susceptibility test for chronic periodontitis in Maharashtrian ethnicity. J Periodontol 77: 1515–1521. 16945028 10.1902/jop.2006.050427

[3] Loos BG, Craandijk J, Hoek FJ, Wertheim-van Dillen PM, van der Velden U (2000) Elevation of systemic markers related to cardiovascular diseases in the peripheral blood of periodontitis patients. J Periodontol 71: 1528–1534. 11063384 10.1902/jop.2000.71.10.1528

[4] Shi D, Meng H, Xu L, Zhang L, Chen Z, Feng X, et al (2008) Systemic inflammation markers in patients with aggressive periodontitis: a pilot study. J Periodontol 79: 2340–2346. 19053925 10.1902/jop.2008.080192

[5] Holla LI, Fassmann A, Stejskalova A, Znojil V, Vanek J, et al (2004) Analysis of the interleukin-6 gene promoter polymorphisms in Czech patients with chronic periodontitis. J Periodontol 75: 30–36. 15025214 10.1902/jop.2004.75.1.30

[6] Nibali L, Donos N, Brett PM, Parkar M, Ellinas T, et al (2008) A familial analysis of aggressive periodontitis–clinical and genetic findings. J Periodontal Res 43: 627–634. 18752567 10.1111/j.1600-0765.2007.01039.x

[7] Laine ML, Loos BG, Crielaard W. (2010) Gene polymorphisms in chronic periodontitis. Int J Dent 324719. 10.1155/2010/324719 PMC2844543 20339487

[8] Woods A, Brull DJ, Humphries SE, Montgomery HE (2000) Genetics of inflammation and risk of coronary artery disease: the central role of interleukin-6. Eur Heart J 21: 1574–1583. 10988009 10.1053/euhj.1999.2207

[9] Castell JV, Gómez-Lechón MJ, David M, Andus T, Geiger T, et al (1989) Interleukin-6 is the major regulator of acute phase protein synthesis in adult human hepatocytes. FEBS Lett 242: 237–239. 2464504 10.1016/0014-5793(89)80476-4

[10] Offenbacher S, Odle BM, Braswell LD, Johnson HG, Hall CM, et al (1989) Changes in cyclooxygenase metabolites in experimental periodontitis in Macaca mulatta. J Periodontal Res 24: 63–74. 2524572 10.1111/j.1600-0765.1989.tb00859.x

[11] Loo WT, Wang M, Jin LJ, Cheung MN, Li GR (2011) Association of matrix metalloproteinase (MMP-1, MMP-3 and MMP-9) and. Cyclooxygenase-2 gene polymorphisms and their proteins with chronic periodontitis. Arch Oral Biol 56: 1081–1090. 21481333 10.1016/j.archoralbio.2011.03.011

[12] Dubois RN, Abramson SB, Crofford L, Gupta RA, Simon LS, et al (1998) Cyclooxygenase in biology and disease. FASEB J 12: 1063–1073. 9737710

[13] Tipton DA, Flynn JC, Stein SH, Dabbous MKh (2003) Cyclooxygenase-2 inhibitors decrease interleukin-1beta-stimulated prostaglandin E2 and IL-6 production by human gingival fibroblasts. J Periodontol 74: 1754–1763. 10.1902/jop.2003.74.12.1754 14974816

[14] Nikolopoulos GK, Dimou NL, Hamodrakas SJ, Bagos PG (2008) Cytokine gene polymorphisms in periodontal disease: a meta-analysis of 53 studies including 4178 cases and 4590 controls. J Clin Periodontol 35: 754–767. 18673406 10.1111/j.1600-051X.2008.01298.x

[15] Fishman D, Faulds G, Jeffery R, Mohamed-Ali V, Yudkin JS, et al (1998) The effect of novel polymorphisms in the interleukin-6 (IL-6) gene on IL-6 transcription and plasma IL-6 levels, and an association with systemic-onset juvenile chronic arthritis. J Clin Invest 102: 1369–1376. 9769329 10.1172/JCI2629 PMC508984

[16] Bennermo M, Held C, Green F, Strandberg LE, Ericsson CG, et al (2004) Prognostic value of plasma interleukin-6 concentrations and the -174 G>C and -572 G>C promoter polymorphisms of the interleukin-6 gene in patients with acute myocardial infarction treated with thrombolysis. Atherosclerosis 174: 157–163. 15135265 10.1016/j.atherosclerosis.2004.01.019

[17] Fife MS, Ogilvie EM, Kelberman D, Samuel J, Gutierrez A, et al (2005) Novel IL-6 haplotypes and disease association. Genes Immun 6: 367–370. 15815691 10.1038/sj.gene.6364186

[18] Shao MY, Huang P, Cheng R, Hu T (2009) Interleukin-6 polymorphisms modify the risk of periodontitis: a systematic review and meta-analysis. J Zhejiang Univ Sci B 10: 920–927. 19946956 10.1631/jzus.B0920279 PMC2789527

[19] Ho YP, Lin YC, Yang YH, Ho KY, Wu YM, et al (2008) Cyclooxygenase-2 Gene-765 single nucleotide polymorphism as a protective factor against periodontitis in Taiwanese. J Clin Periodontol 35: 1–8. 10.1111/j.1600-051X.2007.01167.x 18173398

[20] Schaefer AS, Richter GM, Nothnagel M, Laine ML, Noack B, et al (2010) COX-2 is associated with periodontitis in Europeans. J Dent Res 89: 384–388. 20177132 10.1177/0022034509359575

[21] Ainamo J, Bay I (1975) Problems and proposal for recording gingivitis and plaque. Dent J 25: 229–235. 1058834

[22] Arbes SJ, Slade GD, Beck JD (1999) Association between extent of periodontal attachment loss and self-reported history of heart attack: an analysis of NHANES lll data. J Dent Res 78: 1777–1782. 10598906 10.1177/00220345990780120301

[23] Nibali L, Pelekos G, D’Aiuto F, Chaudhary N, Habeeb R, et al (2013) Influence of IL-6 haplotypes on clinical and inflammatory response in aggressive periodontitis. Clin Oral Investig 17: 1235–1242. 10.1007/s00784-012-0804-3 22918663

[24] Nibali L, Donos N, Farrell S, et al (2010) Association between interleukin-6–174 polymorphism and Aggregatibacter actinomycetemcomitans in chronic periodontitis. J Periodontol 81: 1814–1819. 20681812 10.1902/jop.2010.100084

[25] Nibali L, D′Aiuto F, Donos N, Ready D, Pratten J, et al (2009) Association between periodontitis and common variants in the promoter of the interleukin-6 gene. Cytokine 45: 50–54. 19084430 10.1016/j.cyto.2008.10.016

[26] Trevilatto PC, Scarel-Caminaga RM, de Brito RB Jr, de Souza AP, Line SR (2003) Polymorphism at position −174 of IL-6 gene is associated with susceptibility to chronic periodontitis in a Caucasian Brazilian population. J Clin Periodontol 30: 438–442. 12716337 10.1034/j.1600-051x.2003.20016.x

[27] Jansson H, Lyssenko V, Gustavsson A, Hamberg K, Söderfeldt B, et al (2006) Analysis of the interleukin-1 and interleukin-6 polymorphisms in patients with chronic periodontitis. A pilot study. Swed Dent J 30: 17–23. 16708852

[28] Kalburgi N, Bhatia A, Bilichodmath S, Patil S, Mangalekar S, et al (2010) Interleukin-6 promoter polymorphism (-174 G/C) in Indian patients with chronic periodontitis. J Oral Sci 52: 431–437. 20881337 10.2334/josnusd.52.431

[29] Mesa F, Aguilar M, Galindo_Moreno P, Bravo M, O’Valle F (2012) Cox-2 Expression in Gingival Biopsies From Periodontal Patients is Correlated With Connective Tissue Loss. J Periodontol 83: 1538–1545. 22324469 10.1902/jop.2012.110561

[30] Morton RS, Dongari-Bagtzoglou AI (2001) Cyclooxygenase-2 is upregulated in inflamed gingival tissues. J Periodontol 72: 461–9. 11338298 10.1902/jop.2001.72.4.461

[31] Xie CJ, Xiao LM, Fan WH, Xuan DY, Zhang JC (2009) Common single nucleotide polymorphisms in cyclooxygenase-2 and risk of severe chronic periodontitis in a Chinese population. J Clin Periodontol 36: 198–203. 19236532 10.1111/j.1600-051X.2008.01366.x

[32] Papafili A, Hill MR, Brull DJ, McAnulty RJ, Marshall RP, et al (2002) Common promoter variant in cyclooxygenase-2 represses gene expression: evidence of role in acute-phase inflammatory response. Arterioscler Thromb Vasc Biol 22: 1631–1636. 12377741 10.1161/01.atv.0000030340.80207.c5

[33] Hill MR, Papafili A, Booth H, Lawson P, Hubner M, et al (2006) Functional prostaglandin-endoperoxide synthase 2 polymorphism predicts poor outcome in sarcoidosis. Am J Respir Crit Care Med 174: 915–922. 16840740 10.1164/rccm.200512-1839OC

[34] Ol KK, Agachan B, Gormus U, Toptas B, Isbir T (2011) Cox-2 gene polymorphism and IL-6 levels in coronary artery disease. Genet Mol Res 10: 810–6. 21574137 10.4238/vol10-2gmr967

